# Waist-to-Height Ratio Percentiles and Cutoffs for Obesity: A Cross-sectional Study in Brazilian Adolescents

**Published:** 2014-09

**Authors:** Isa de Pádua Cintra, Maria Aparecida Zanetti Passos, Luana Caroline dos Santos, Helymar da Costa Machado, Mauro Fisberg

**Affiliations:** ^1^Adolescent Sector, Department of Pediatrics, Universidade Federal de São Paulo, São Paulo, SP, Brazil; ^2^Department of Nutrition, School of Nursing, Universidade Federal de Minas Gerais, Belo Horizonte, MG, Brazil; ^3^Statistics Division, Universidade Estadual de Campinas, Brazil

**Keywords:** Adolescents, Central adiposity, Obesity, Waist-to-height ratio, Brazil

## Abstract

This study aimed to describe the distribution of waist-to-height ratio (WHtR) percentiles and cutoffs for obesity in Brazilian adolescents. A cross-sectional study including adolescents aged 10 to 15 years was conducted in the city of São Paulo, Brazil; anthropometric measurements (weight, height, and waist-circumference) were taken, and WHtRs were calculated and then divided into percentiles derived by using Least Median of Squares (LMS) regression. The receiver operating characteristic (ROC) curve was used in determining cutoffs for obesity (BMI ≥97th percentile) and Mann-Whitney and Kruskal-Wallis tests were used for comparing variables. The study included 8,019 adolescents from 43 schools, of whom 54.5% were female, and 74.8% attended public schools. Boys had higher mean WHtR than girls (0.45±0.06 vs 0.44±0.05; p=0.002) and higher WHtR at the 95th percentile (0.56 vs 0.54; p<0.05). The WHtR cutoffs according to the WHO criteria ranged from 0.467 to 0.506 and 0.463 to 0.496 among girls and boys respectively, with high sensitivity (82.8-95%) and specificity (84-95.5%). The WHtR was significantly associated with body adiposity measured by BMI. Its age-specific percentiles and cutoffs may be used as additional surrogate markers of central obesity and its co-morbidities.

## INTRODUCTION

The appearance of secondary sexual characteristics, growth spurt and changes in body composition during adolescence vary greatly between individuals, making it difficult to establish specific criteria for classification of nutritional status, especially based on chronological age (1).

According to the World Health Organization (WHO) (2), nutritional status can be assessed by comparing observed measures with normal values obtained in a reference population, which reflect normal growth of a population under optimal health and nutritional conditions.

Anthropometric indices are usually used in determining nutritional status based on standards established from data of population-based studies. The most common anthropometric indices are: weight-for-height, height-for-age, body mass index (BMI), waist-to-hip ratio (WHR) and, more recently, waist-to-height ratio (WHtR) (2,3).

The WHtR is a simple and practical measure, and researchers have suggested that WHtR might be a better predictor of the risk of cardiovascular disease than BMI or waist-circumference (WC) (4). WHtR is more strongly correlated with visceral fat-mass (5) and clustering of cardiovascular risk factors in children (6) and adults (7). It incorporates WC as a measure of abdominal adiposity and adjusts for an individual's size by dividing by their height. The use of WHtR has been proposed as it may explain the metabolic consequences of obesity and identify abdominal obesity, particularly in individuals who would not be classified as overweight or obese by BMI (8-12). WHtR has no measurement units and is in close agreement in both males and females in an age-group. However, Tybor *et al.* (13) has claimed that simply dividing WC by height might not adequately “adjust for height” during periods of growth. To minimize residual correlation with height, the exponent for height in a waist-to-height index would need to be sex- and age-specific (6-18).

The pattern of regional distribution of body-fat is concerning because there is an intrinsic relationship between fat accumulation and development of metabolic disorders, such as insulin resistance, dyslipidaemia, diabetes, and other non-communicable diseases (19-22).

In Brazil, recent data revealed that malnutrition in young population co-exists with alarming high prevalence of overweight and obesity, which has an impact on the national healthcare system and other cultural, social, and economic effects. Data from the POF (Pesquisa de Orçamentos Familiares)-Brazilian National Data of Budget Familial Research (2008-2009) provided by the Brazilian Institute of Geography and Statistics showed that 21.7% of male and 19.4% of female adolescents are overweight (23).

In light of that, the present study aimed to assess WHtR among Brazilian adolescents and to propose cutoffs according to the percentiles of body adiposity in this population.

## MATERIALS AND METHODS

### Study design and sampling

The study population was drawn from a prior survey titled, “Nutritional profile of adolescents from public and private schools in São Paulo”, a segmented population-based cross-sectional study with anthropometric data and other information collected using questionnaire. Schools were selected based on the 2002 School Census, which included all schools from four major areas of the city of São Paulo: North, Midwest, East, and South.

Local offices were contacted and asked to provide information on schools with students aged 10 to 15 years, attending morning and afternoon classes, their locations, and number of students enrolled. The total number of schools by area was ascertained, and the proportional relationship between public and private schools was calculated taking into account the number of schools by area, i.e. areas with more schools would have a larger number of schools assessed. The following exclusion criteria were applied in selection of schools: schools with students aged 10 to 16 years attending night classes only; schools with difficult access and/or located in violent areas; and schools with a small number of students (less than 200). The remaining schools were then randomly selected; in case of refusal, another school in the same area was drawn. All principals of the participating schools signed a consent form.

There were 43 schools under study in the city of São Paulo: 32 public and 11 private. These were located in the four major areas of the city as follows: 17% in the North, 17% in the Midwest, 37% in the East, and 29% in the South. The largest number of public schools were in the Eastern area of the city (n=11; 34.4%), and the largest number of private schools were in the South (n=6; 54.5%). Their original distribution was preserved. Data were collected between September 2004 and June 2005. Adolescents were excluded if they met any of the following criteria: pregnancy, younger or older than the age-range, and having a physical condition that would prevent routine anthropometric assessment. No formula was used in estimating the sample-size. A probabilistic approach was followed, and all selected schools that agreed to participate in the study were asked to give the students a free informed consent form to be signed by their parents or guardians agreeing to their children's participation. Only students who returned a signed consent form were included in the study.

All ethical principles of Resolution 196 of the National Brazilian Health Council were followed, and the study was approved by the Research Ethics Committee of Universidade Federal de São Paulo (No. 0977/03).

### Study protocol

The anthropometric assessment was led by a team comprising four researchers, three nutritionists, and a physical educator—all of them were graduate students properly trained in the required techniques and standard procedures. This same team conducted a pilot study with more than 2,000 adolescents and calculated intra- and inter-observer reliability to minimize errors.

Information on demographic and anthropometric variables and pubertal stage was obtained. Demographic information (age and gender) was collected through a pretested questionnaire administered in a face-to-face interview. A self-assessment method was used in determining sexual maturation, using Tanner's pubertal staging for breast development (B1, B2, B3, B4, B5) in girls and genitalia (G1, G2, G3, G4, G5) in boys (24). The adolescents were instructed on the self-assessment following the WHO recommendations (2).

The anthropometric assessment included weight, height, and waist-circumference (WC) measurements following the proposed techniques (2). Weight was measured using a digital portable scale (Seca^®^) with capacity of 150 kg, and height was measured using a wall-mounted digital stadiometer (Seca^®^). WC measures were taken preferably at midpoint between the costal margin and iliac crest (25), using an inelastic metric tape (Seca^®^).

All anthropometric measurements were taken during science and physical education classes. Adolescents’ self-assessment of sexual maturation was performed in a separate room at school. The very school administration would establish a schedule for evaluation days so that it would not interfere with daily school routines. School teachers helped dealing with the students during assessments.

BMI—defined as weight in kg divided by height in metres squared (m^2^)—was calculated and classified according to WHO criteria (26): underweight: BMI <3th percentile; normal weight: 3th ≤BMI ≤85th; overweight: 85th <BMI ≤97th; and obesity: BMI> 97th.

The WHtR was obtained by dividing WC (cm) by height (cm).

### Data analysis

Smoothed WHtR centile curves were constructed by the LMS method (27,28), using the LMS Chartmaker Pro (version 2.3) (The Institute of Child Health, London).

To verify the normality of WHtR values, the Kolmogorov-Smirnov test (p<0.05) and the coefficients of skewness and kurtosis were used. They all showed a significant departure from normality and, therefore, a non-parametric statistical analysis was performed. A descriptive analysis of the study variables was carried out, and Mann-Whitney test was used for comparing WHtRs between the two groups and the Kruskal-Wallis test for comparing percentiles (three or more groups).

Additionally, an analysis was performed using the receiver operating characteristic (ROC) curve for WHtR to assess sensitivity and specificity of cutoffs according to BMI classification based on WHO criteria.

All analyses were performed using Statistical Analysis System (SAS) package (29) at 5% significance level.

## RESULTS

A total of 8,019 adolescents participated in the study, of whom 54.5% (4,371) were female, with mean age of 13.04 (1.27) years. In the study, girls were younger than boys [13.03 (1.26) vs 13.06 (1.29) years old; p=0.002].

The sample comprised 2.23% of 360,000 middle school students enrolled in public and private schools in the city of São Paulo.

With regard to nutritional status, 69.5% of the adolescents studied were of normal weight, 17.2% overweight, 10.3% obese, and 0.65% extremely obese. Excess weight was more prevalent in adolescent males than females (31.6% vs 25.4%) and among the private compared to the public school students (36.6% vs 25.3%). There was no statistical difference in nutritional disorders by city area (North, South, East, and Midwest) (p=0.097), with the lowest prevalence of excess weight in the East (27.4%) and the highest prevalence in the South (28.6%).

A higher mean WHtR was found among males than females [0.45 (0.06) vs 0.44 (0.05); p=0.002]. In addition, 27.6% of girls and 33.5% of boys were in early pubertal stages (1–2). WHtR increased in girls with sexual maturation: 0.43 (0.06)–B1; 0.43 (0.05)–B2; 0.44 (0.05)–B3; 0.46 (0.06)–B4; and 0.47 (0.06)–B5 (p<0.001); it decreased in boys with sexual maturation: 0.46 (0.06)–G1; 0.45 (0.06)–G2; 0.44 (0.05)–G3; 0.44 (0.06)–G4; 0.43 (0.06)–B5. Statistically different WHtRs were seen in the following stages of puberty in girls: B1≠(B4 and B5); B2≠(B4 and B5); B3≠(B4 and B5); and in boys: G1≠G5. The highest WHtR values were seen in two girls in B3 (0.72), one in B5 (0.71), and in two boys in G2 (0.71 and 0.83). Higher mean WHtRs were seen in menarche compared to non-menarche girls [0.44 (0.05) vs 0.43 (0.05), p<0.001].

There was a linear increase in WHtRs between BMI percentiles with the following distribution in adolescent females: <3th: 0.377±0.032; 5th-85th: 0.421±0.035; 85th-97th: 0.482±0.040; >97th: 0.536±0.059 (p<0.001); and in adolescent males: <3th: 0.386±0.041; 5th-85th: 0.419±0.029; 85th-97th: 0.478±0.038; >97th: 0.544±0.055 (p<0.001).

The highest mean WHtRs were seen at age 11 years in boys and age 15 years in girls (Table 1). Significant differences in age in terms of WHtRs were found in adolescent females as follows: 10≠15, 12≠15, and 13≠15 years; and in males: 10≠13; 10≠14; 10≠15; 11≠13; 11≠14; 11≠15; 12≠14; and 12≠15 years.

Table 2 and 3 show WHtR percentile distribution in adolescent females and males.

WHtR cutoffs by age and gender showed high sensitivity (82.8-91.7% and 89.2-95% in females and males respectively) and high specificity (84.0-94.1% and 89.2-95.5% in females and males respectively) based on WHO criteria for obesity (Table 4). Central obesity was seen in 18.57% of girls and 20.96% of boys based on the cutoffs proposed in this study. The prevalence of overweight based on WHO cutoffs (26) was 19.6% and 24.1% in girls and boys respectively, in the sample studied. Of them, 56.1% were 10-12 years old.

**Table 1.** Mean waist-to-height ratios by age and gender

Gender	Age^a^	n	Mean	SD	Minimum	Median	Maximum	p value
Female	10	180	0.437	0.055	0.330	0.424	0.679	p<0.001
	11	891	0.440	0.053	0.295	0.430	0.644	
	12	999	0.438	0.056	0.329	0.428	0.662	
	13	1,106	0.439	0.053	0.331	0.427	0.717	
	14	961	0.444	0.055	0.341	0.435	0.710	
	15	234	0.445	0.048	0.325	0.435	0.632	
Male	10	134	0.454	0.054	0.380	0.437	0.617	p<0.001
	11	765	0.458	0.060	0.269	0.443	0.677	
	12	816	0.449	0.059	0.259	0.431	0.717	
	13	904	0.443	0.061	0.224	0.426	0.831	
	14	787	0.435	0.050	0.346	0.422	0.620	
	15	242	0.432	0.056	0.347	0.419	0.678	

^a^Indicates whole-year age-group, e.g. 10.0-10.99 years, etc.; SD=Standard deviation

**Table 2.** Smoothed age-specific waist-to-height ratio percentiles for adolescent females aged 10-15 years

Age (completed years)	3rd	5th	10th	15th	25th	50th	75th	85th	90th	95th	97th
10	0.36	0.37	0.38	0.39	0.40	0.43	0.47	0.50	0.52	0.55	0.57
11	0.36	0.37	0.38	0.39	0.41	0.44	0.47	0.50	0.51	0.54	0.57
12	0.37	0.37	0.38	0.39	0.41	0.44	0.47	0.50	0.52	0.55	0.58
13	0.36	0.37	0.38	0.39	0.41	0.44	0.47	0.50	0.52	0.55	0.58
14	0.36	0.37	0.38	0.39	0.41	0.44	0.47	0.50	0.51	0.54	0.56
15	0.37	0.38	0.39	0.40	0.41	0.44	0.48	0.50	0.51	0.54	0.56

**Table 3.** Smoothed age-specific waist-to-height ratio percentiles for adolescent males aged 10-15 years

Age (completed years)	3rd	5th	10th	15th	25th	50th	75th	85th	90th	95th	97th
10	0.39	0.40	0.41	0.42	0.43	0.46	0.50	0.53	0.55	0.60	0.64
11	0.38	0.38	0.40	0.40	0.42	0.45	0.49	0.52	0.54	0.58	0.60
12	0.36	0.37	0.38	0.39	0.41	0.45	0.49	0.51	0.53	0.57	0.59
13	0.37	0.37	0.38	0.39	0.41	0.44	0.48	0.51	0.53	0.56	0.59
14	0.37	0.38	0.39	0.40	0.41	0.44	0.47	0.50	0.52	0.55	0.58
15	0.37	0.38	0.39	0.40	0.41	0.44	0.47	0.50	0.52	0.56	0.59

**Table 4.** Sensitivity and specificity of waist-to-height ratio cutoffs by gender and age according to the receiver operating characteristic (ROC) curve analysis for obesity based on the World Health Organization (WHO) criteria

Gender	Age (completed years)	Sample (n)	Area*	95% CI of the area**	Sensitivity (%)	Specificity (%)	WHtR
Female	10	180	0.911	0.844-0.979	91.3	87.3	≥0.467
11	891	0.889	0.844-0.934	82.8	85.5	≥0.475
12	999	0.933	0.906-0.960	91.2	84.0	≥0.474
13	1,106	0.936	0.909-0.963	89.7	86.4	≥0.479
14	961	0.945	0.917-0.973	87.5	90.5	≥0.503
15	234	0.967	0.927-1.000	91.7	94.1	≥0.506
Male	10	134	0.960	0.926-0.993	92.0	90.8	≥0.483
11	765	0.934	0.908-0.961	89.2	89.5	≥0.489
12	816	0.937	0.914-0.960	89.8	86.1	≥0.480
13	904	0.943	0.916-0.969	89.4	91.8	≥0.489
14	787	0.979	0.970-0.988	94.7	95.5	≥0.496
15	242	0.942	0.865-1.000	95.0	89.2	≥0.463

*Area under the ROC curve;

**95% CI=95% confidence interval of the area under the ROC curve; WHtR=Waist-to-height ratio

The analysis of WHtR cutoffs by gender showed lower sensitivity (84.2% vs 88.6% in girls and 85.7 vs 88.8 in boys) and lower specificity for overweight than obesity (77.0 vs 83.3 in girls and 80.8 vs 91.5 in boys) based on WHO criteria (Table 5).

## DISCUSSION

This study assessed the distribution of WHtR percentiles and cutoffs that would more likely identify adiposity in adolescents aged 10-15 years in the city of São Paulo. The WHtR is regarded as independent of age and gender (30). However, we found statistically significant differences in WHtR in our study population by gender (higher values among adolescent males) and age (11≠13, 15 years).

Weili *et al.* (31) reported in Chinese children and adolescents aged 8 to 18 years cutoffs of 0.445 for overweight in both females and males and cutoffs for obesity of 0.475 in females and 0.485 in males. Although these authors (31) have studied a population with a larger age-range and different biotypes, our WHtRs to identify overweight in young populations were very close. This finding suggests that children and adolescents with these WHtRs may be at risk of adiposity.

It is noteworthy that significant differences in WHtRs were found between boys and girls, findings corroborating other authors (32-34). Sung *et al.* (35) found significant differences between age-groups and reported age-specific WHtRs, ranging from 0.47 to 0.42 among boys and from 0.46 to 0.41 among girls, which are close to those reported in our study. It suggests that the use of age-specific cutoffs may provide more reliable assessments.

The differences in WHtR found in our study are likely to be due to differences in overall adiposity; 31.6% of boys were overweight or obese compared to 25.4% of girls. The different cutoffs in different stages of sexual maturation are noteworthy, pointing to an interaction with physical changes during adolescence and a need for using different cutoffs. Guntsche *et al.* (36) evaluated Spanish children and adolescents aged 6-16 years and found no differences in WHtR cutoffs by pubertal stage but found a small difference between mean WHtR values in lean boys and girls. They stressed that they measured umbilical WC; however, similar results were not found with WC measures based on WHO recommendations.

A limitation of our study was the age-range studied. Most students were pubescent. Of the 8,019 adolescents studied, sexual maturation was assessed in 90.1% of the sample (7,226, 54.8% of females) because some schools did not allow the self-assessment of their students. Of the adolescent females and males evaluated, 3.7% and 4.4% were in stage 1, 26.7% and 33.1% in stage 2; 45.6% and 41.1% in stage 3; 20.65% and 20.5% in stage 4; and 3.3% and 0.9% in stage 5. Excess weight was seen in 16.9% of females in B1, 19.9% in B2, 23% in B3, 35.8% in B4, and 43.2% in B5. Higher prevalence of overweight was seen in stage B4 and B5; a difference in WHtRs was also seen in these stages compared to all other pubertal stages. Excess weight was observed in 36.1% of males in G1, 29.4% in G2, 29% in G3, 30.3% in G4, and 25% in G5—a difference in WHtR by sexual maturity that was only statistically significant between G1 (higher prevalence of excess weight) and G5 (lower prevalence of excess weight).

**Table 5.** Optimal waist-to-height ratio cutoffs for identifying overweight and obesity and their related sensitivity and specificity in Brazilian adolescent females and males aged 10-15 years

Gender	Age (completed years)	Sample (n)	Area*	95% CI of the area**	Sensitivity (%)	Specificity (%)	WHtR
Overweight							
Girls	10-15	4,371	0.878	0.865-0.891	84.2	77.0	≥0.443
Boys	10-15	3,648	0.907	0.894-0.919	85.7	80.8	≥0.439
Obesity							
Girls	10-15	4,371	0.920	0.903-0.937	88.6	83.3	≥0.475
Boys	10-15	3,648	0.948	0.936-0.959	88.8	91.5	≥0.489

*Area under the ROC curve;

**95% CI=95% confidence interval of the area under the ROC curve; WHtR=Waist-to-height ratio

Guntsche *et al.* (36) reported that the difference in WHtR by gender was no longer seen in those with excess weight, which further supports the use of this measure in children and adolescents with excess body-weight. It should be noted that, while excess weight was seen in 64% of participants in the study by Guntsche *et al.* (36), it was found in 28.15% in our study—a prevalence that is higher than that reported in a study that evaluated Brazilian adolescents. Further investigations are needed to assess more accurately the impact of sexual maturation on this measure of adiposity in growing adolescents.

The WHtR cutoffs that were proposed in the present study based on BMI percentiles associated with body adiposity showed high sensitivity and specificity. Ashwel *et al.* (37) proposed a universal WHtR cutoff of 0.5, which is close to the age-specific cutoffs ranging from 0.467 to 0.506 determined in the present study.

According to Goulding *et al.* (38), there is a curvilinear relationship between BMI and WHtR, and 90% of children and adolescents with BMI above the 95th percentile had WHtR ≥0.5. The use of this cutoff would allow identifying cardiovascular risk in these groups. However, when this cutoff was used, central obesity was identified in only 13.01% and 16.67% of girls and boys respectively.

Ying-Xiu *et al.* (17) evaluated 42,296 students aged 7-18 years from Shandong and found WHtR ≥0.5 in 7.38% of girls and 15.73% of boys—a prevalence that is lower than that found in the present study. Xiong *et al.* (34) evaluated Chinese children and adolescents and found WHtR ≥0.5 in 10.8% and 14.8% of girls and boys respectively while 16.4% and 26.4% of them were overweight based on the International Obesity Task Force (IOTF) cutoffs.

It was found that 8.5% of girls and 14.1% of boys were obese (BMI >97th percentile), with mean WHtR of 0.536 and 0.544 respectively. The highest WHtRs—0.83 and 0.72—were seen in boys and girls aged 13 years with BMI of 54.7 and 32.97 respectively. If the WHtR cutoffs were ≥0.5, 75% of obese girls and 88.4% of obese boys would be at risk; however, using the cutoffs proposed in the present study (0.475 for females and 0.489 for males), 88% of obese girls and 88.4% of obese boys would have abdominal adiposity. Only 1.8% of adolescents with a normal BMI category had a WHtR ≥0.5. Conversely, the majority of obese adolescents had an elevated WHtR.

Bearing in mind that this condition is also present in adolescents with normal weight, we believe that the proposed WHtR cutoffs are the most suitable tool for assessing central obesity in Brazilian adolescents.

Other authors reported different WHtR cutoffs. Panjikkaran (18) evaluated Indian school children at risk of excess weight and found that the area under the ROC curve was 0.827 at WHtR 0.48 compared to 0.673 at WHtR 0,50, showing that 0.48 is an optimal cutoff for this population. Motswagole *et al.* (39) found in children and adolescents aged 9-15 years that a WHtR cutoff of 0.41 was adequate to identify hypertension in both girls and boys. The Australian Health and Fitness Survey 1985 evaluated 2,773 subjects aged 8-16 years and found WHtRs of 0.46 and 0.48 in boys and 0.45 and 0.47 in girls at the 85th and 95th percentile of body-fat respectively. The highest WHtRs were associated with the highest mean serum lipid levels and blood pressure (40). A WHtR cutoff of 0.5 might identify children and adolescents at metabolic risk but, as the present study focused on the risk of adiposity, further investigation is warranted.

Abdominal fat, regardless of whether an individual is overweight or not, is a major risk factor of the aforementioned conditions, especially during adolescence. Excess weight gain and the resulting changes in body composition may lead to metabolic changes at this stage and future stages of life (41-49).

Growing evidence shows that central fat excess tends to increase with age, starting during the growth process of childhood and adolescence and extending throughout adult age. WHtR seems an effective, straightforward indicator for measuring abdominal obesity in both children and adults and more suitable to identify coronary risk than BMI and WC (50,51).

A study on 38,556 residents of Taiwan found that WHtR was significantly associated with cardiovascular risk factors, such as hypertension, glucose intolerance, diabetes, and dyslipidaemia (52). However, similar studies in children and adolescents are scarce.

Freedman *et al.* (51) evaluated 2,498 children and adolescents aged 5 to 17 years and found a strong correlation between WHtR and visceral fat and risk factors of cardiovascular disease. Furthermore, Mokha *et al.* (32) found in children and adolescents aged 4-18 years that WHtR is not only an important indicator of central obesity and adverse cardiometabolic risk for obese children but also for normal-weight children with WHtR >0.5. They found central obesity in 9.2% of children with normal weight and adolescents with WHtR ≥0.5; a multivariate analysis showed they were 1.66, 2.01, 1.47, and 2.5 times more likely to have abnormal plasma LDL-cholesterol, HDL-cholesterol, triglyceride, and insulin levels. Those with a family history of type 2 diabetes had a high prevalence of metabolic syndrome.

Regarding cardiometabolic risk, Elizondo-Montemayor *et al.* (53) found that a cutoff of 0.59 from the ROC curve was identified as a strong predictor of metabolic syndrome in Mexican children aged 6-12 years.

Brambilla *et al.* (16) reported that WHtR is better than WC and BMI for predicting adiposity in children and adolescents and can be a valuable tool for assessing body-fat when skinfold measurements are not available. Burns *et al.* (54) also demonstrated that this measure may be preferable to BMI in identifying children with suboptimal physical fitness, which reinforces the importance of WHtR as a measure of body adiposity.

### Limitations

A limitation of this study is that BMI for age and gender was used as the gold standard to test WHtR cutoffs while the use of body-composition measures could provide more accurate results. However, the original study had a large sample, and these indicators were not evaluated in the entire population, which prevented further comparisons.

### Conclusions

WHtR cutoffs for overweight and obesity were analyzed by gender, and it showed lower sensitivity and specificity, especially for overweight. However, these cutoffs are useful in population-based studies, and the cutoff values found for obesity were quite similar to those reported in the literature.

The present study showed that WHtR was significantly associated with body adiposity and BMI. Age-specific WHtR percentiles and cutoffs may be used as surrogate markers of central obesity and its co-morbidity and is, therefore, an important tool for additional clinical assessment in Brazilian adolescents.
